# Potential therapeutic effects of branched-chain amino acids supplementation on resistance exercise-based muscle damage in humans

**DOI:** 10.1186/1550-2783-8-23

**Published:** 2011-12-14

**Authors:** Claudia R da Luz, Humberto Nicastro, Nelo E Zanchi, Daniela FS Chaves, Antonio H Lancha

**Affiliations:** 1 Laboratory of Applied Nutrition and Metabolism, School of Physical Education and Sports, University of São Paulo, São Paulo, SP, Brazil; 2 Institute of Biomedical Science, University of São Paulo, São Paulo, SP, Brazil

**Keywords:** Leucine, Delayed onset muscle soreness, Creatine kinase, Protein turnover

## Abstract

Branched-chain amino acids (BCAA) supplementation has been considered an interesting nutritional strategy to improve skeletal muscle protein turnover in several conditions. In this context, there is evidence that resistance exercise (RE)-derived biochemical markers of muscle soreness (creatine kinase (CK), aldolase, myoglobin), soreness, and functional strength may be modulated by BCAA supplementation in order to favor of muscle adaptation. However, few studies have investigated such effects in well-controlled conditions in humans. Therefore, the aim of this short report is to describe the potential therapeutic effects of BCAA supplementation on RE-based muscle damage in humans. The main point is that BCAA supplementation may decrease some biochemical markers related with muscle soreness but this does not necessarily reflect on muscle functionality.

## Introduction

Skeletal muscle damage is a phenomenon that can occur due to several factors, such as rupture and/or cell necrosis, representing about 10-55% of total muscular injuries [1]. The main feature of skeletal muscle damage without cell necrosis is the disruption of muscle fibers, specifically the sheath of basal lamina [1]. Regarding mechanical stimuli, specifically resistance exercise (RE), it is known that it can promote microdamage in muscle fibers imposed by contractions and/or overload and, according to the intensity, length, and volume the severity and degree of damage and discomfort may be compounded over time and persist chronically [2]. As functional consequence, muscle damage is manifested by a temporary decrease in strength, increased muscle passive tension, delayed onset muscle soreness (DOMS), and edema [2].

In this context, some prophylactic interventions have been proposed in order to attenuate the negative effects associated with RE-induced muscle damage. Among the nutritional strategies, supplementation with branched-chain amino acids (BCAA - leucine, isoleucine, and valine) has been considered a potential intervention [3,4]. It is known that these amino acids, particularly leucine, have anabolic potential by stimulating the initiation of protein translation [5], possibly suppressing/attenuating muscle proteolysis [6], and offering its transamination product alpha-ketoisocaproate (α-KIC), widely known to inhibit the enzymatic activity of the branched-chain alpha-keto dehydrogenase complex (BCKDH) which increases BCAA oxidation [7,8]. Thus, BCAA supplementation could promote interesting effects on muscle repair by reducing protein oxidation, promoting muscle sarcomerogenesis, and improving muscle functional status.

The purpose of this short review is to describe the effects of BCAA supplementation on RE-induced muscle damage. To this, we considered only human studies since they can elucidate a possible nutritional strategy with therapeutic potential. This strategy may promote benefits such as attenuate muscle soreness and improve skeletal muscle turnover to subjects engaged on resistance exercise program which could favor RE-induced training adaptations. To this end, this report discusses the basic concepts of muscle damage and its biochemical markers followed by evidences of effects of BCAA supplementation on RE-induced muscle damage in humans.

## Discussion

### Cellular responses and biochemical markers of muscle damage

The damage of muscle tissue can be defined as the disruption of plasma membrane accompanied by the loss of muscle proteins (i.e. creatine kinase (CK), myoglobin, lactate dehydrogenase (LDH), aldolase, troponin), the influx of serum proteins, increased population of inflammatory infiltrates in the muscle fibers (i.e. macrophages and neutrophils), DOMS, functional impairment (strength loss), and possible structural disorders such as sarcomere Z lines disarrangement [9,10]. Current literature classifies the damage of skeletal muscle in two stages called primary and secondary damage [2]. The primary damage can be subdivided into two possible mechanisms: metabolic and mechanical. The metabolic damage has been proposed as a result of ischemia or hypoxia during prolonged exercise, which may results in changes in ion concentration, accumulation of metabolic wastes, and deficiency of adenosine triphosphate (ATP) [11]. Mechanical stimuli, however, may induce muscle damage as direct consequence of overload of muscle fibers or inappropriate balance of exercise variables that can cause the disruption of the sarcomeric Z lines [2], [9,10]. The secondary damage can be manifested through processes associated with exercise that can lead to disruption of intracellular calcium homeostasis and systemic and local inflammatory response [11]. Of note, it has been proposed that RE-induced muscle damage may be a necessary step to favor muscle remodeling and adaptation [12]. However, chronic muscle damage may delay muscle recovery, functionality, and impair protein turnover [13,14].

Enzymatic skeletal muscle proteins such as CK, LDH, myoglobin, and myosin heavy chain (MHC) may spill from muscle cells to the serum and be used as quantitative markers of cellular damage and recovery [15]. RE may affect the structure of skeletal muscle cells at the sarcolemma and Z disks resulting in increased serum levels of CK. Of note, the time-release and clearance of CK depends mainly on the type of exercise and its variables (volume, intensity, and duration). Apparently, this relationship is mainly dependent of intensity and volume [16]. When exercise intensity is mild/moderate and with low volume, muscle tissue does not undergo significant changes in membrane permeability [17,18]. However, with high intensity and low/moderate volume or low/moderate intensity and with high volume, changes in membrane permeability and increase in serum CK and the enzymes mentioned above may occur. Importantly, serum CK concentration has been associated with muscle functional properties such as strength impairment and reduced ATP resynthesis [9,19,20]. LDH is also a widely used marker of cellular damage. It is already known that mechanical stimuli can induce significant increase of serum LDH and the degree of increase depends on the intensity and duration of the exercise [19,21,22]. This relationship has been demonstrated in several studies [23-25]. Myoglobin is another consistently used biochemical marker of muscle damage. After strenuous exercise, myoglobin is released as a result of degradation of muscle protein structures [19,26,27]. Its serum concentration may be elevated for some days probably due to the low-grade inflammation. The activity of myoglobin has strong correlation with the response of neutrophils induced by stress and is therefore a useful marker for monitoring the integrity of skeletal muscle tissue [19,26,27]. Among the markers mentioned above, most studies observed high inter-subject variability in the activity of CK and LDH in response to RE-induced muscle damage [21,22,28]. In contrast, myoglobin appears to be more applicable since their variability is reduced when compared to the others [27].

### BCAA supplementation and RE-induced muscle damage: results of human studies

Recent studies suggest that BCAA supplementation may improve the repair of RE-induced damaged muscle tissue. Shimomura *et al*. [29] assessed serum free amino acids concentration in young untrained women supplemented with BCAA (5.5 g BCAA in 1.0 g of green tea) 15 minutes prior to performing a squat exercise (7 sets of 20 repetitions). The authors observed that serum BCAA concentrations were significantly decreased in the placebo group when compared to the supplemented group (2.2-fold higher), suggesting that the exercise protocol induced significant BCAA oxidation and the supplementation prevented such effect. Furthermore, another study from the same group [30] found that BCAA supplementation (5 g) 15 minutes before the same RE protocol reduced the peak time of muscle soreness (2-3 days after exercise) in young women by about 45% when compared to the placebo group (dextrin) and this reduction was significant up to 5 days after exercise. These data demonstrate that RE-induced muscle damage increase BCAA uptake from serum to skeletal muscle in order to be used as energy source and/or participate in translation initiation signaling pathway involved in muscle remodeling. Functionally, this appears to have some consequence in muscle pain.

Concerning the time-frame of supplementation, Nosaka *et al*. [3] evaluated the effects on muscle damage supplementing an amino acid mixture (BCAA-enriched; 60% of essential amino acids) 30 minutes before, immediately after, and 4 days post-exercise (900 actions of arm curl with 1.80 to 3.44 kg of range of workload). No significant differences were observed in the supplemented group 30 minutes before and immediately after exercise regarding muscle soreness and damage indexes. However, subjects who ingested the amino acid mixture during 4 days post-exercise presented reduction of serum CK (from 48 to 96 hours), myoglobin (from 24 to 96 hours), and of muscle soreness (from 24 to 96 hours) when compared with the placebo group. However, although no significant differences were observed between groups in isometric maximal voluntary contraction, range of motion, upper arm circumference, and muscle discomfort were decreased up to 4 days after exercise in the supplemented group. These results demonstrate that BCAA supplementation may attenuate muscle soreness and this can be related with some biochemical markers. However, since no results were observed in muscle strength we can postulate that the benefits of BCAA supplementation do not involve structural modulation. Similar responses were observed in the study conducted by Sharp & Pearson [31] which supplemented male subjects with BCAA (1.8 g of leucine, 0.75 g of isoleucine, and 0.75 g of valine) during 3 weeks before and 1 week during a high-intensity total-body RE (3 sets of 8 repetitions maximum, 8 exercises) and observed that serum CK was significantly reduced in BCAA supplemented group during and following the exercise protocol.

In a very elegant study, Jackman *et al*. [32] evaluated the effects of BCAA supplementation (3.5 g of leucine, 2.1 g of isoleucine, and 1.7 g of valine; divided in 4 daily doses) on eccentric exercise-induced muscle damage. The main feature of this study was that the subjects remained in dietary control throughout the experimental protocol in order to minimize the possible effects of other nutrients on the cellular and functional responses. In the exercise day (12 sets of 10 repetitions at 120% of concentric 1 repetition maximum), subjects consumed the supplement 30 minutes before, 1.5 hour after, between lunch and dinner, and before bed; on the following 2 days, 4 doses of supplementation given between meals. Serum CK and myoglobin were significantly increased after exercise and remained throughout the test period and BCAA supplementation did not attenuated it. However, muscle soreness increased after exercise and was 64% reduced in BCAA supplemented group when compared to the placebo group. Thus, it appears that BCAA supplementation can also modulate muscle soreness independently of biochemical markers. Such information need to be elucidated by future studies. In practice, these results reinforce the hypothesis that, although BCAA supplementation does not improve muscle function, it can alleviate RE-induced muscle soreness and favor the subject to perform another RE session (the phenomenon called "repeated bout effect").

Table 1 summarizes the main results described in the text.

**Table 1:** Studies investigating the effects of BCAA supplementation of RE-based muscle damage in humans

Study	Exercise Protocol	Supplementation Protocol	Results
Shimomura *et al*. [29]	Squat (7 sets of 20 repetitions)	5.5 g of BCAA within 1.0 g of green tea 15 min before exercise	Attenuation of exercise-induced serum BCAA oxidation.
Shimomura *et al*. [30]	Squat (7 sets of 20 repetitions)	5.0 g of BCAA 15 min before exercise	Reduction of peak time of muscle soreness induced by exercise.
Nosaka *et al.*[3]	900 actions (30 min) of arm curl with 1.80 to 3.44 kg of range of workload	BCAA-enriched amino acid mixture (60% of the essential amino acids)	Reduction of serum CK, myoglobin, and muscle soreness. No differences in isometric MVC.
Sharp & Pearson [31]	Whole body RE (3 sets of 8 RM, 8 exercises)	BCAA (1.8 g of leucine, 0.75 g of isoleucine, and 0.75 g of valine) 3 weeks before and 1 week during exercise protocol	Reduction of serum CK.
Jackman *et al*. [32]	Eccentric exercise (12 sets of 10 repetitions at 120% of concentric 1RM)	~ 7.0 g of BCAA/day (divided in 4 doses) on the following 2 days after exercise	No differences in serum CK and myoglobin; Attenuation in exercise-induced muscle soreness.

BCAA = branched-chain amino acids; CK = creatine kinase; MVC = maximal voluntary contraction; RE = resistance exercise; RM = repetition maximum.

## Conclusions and perspectives

According to the data presented, BCAA supplementation appears to be an interesting nutritional intervention to alleviate RE-induced muscle soreness. Although some studies have found that biochemical markers of muscle damage are reduced after BCAA supplementation, it does not reflect improved muscle function (at least in short term studies). Paradoxically, although RE, especially lengthening (eccentric) contractions, is associated with muscle injury, they can also provide significant protection against future muscle damage and are robustly involved in the hypertrophy process [33]. However, little is known about the conditions that result in the protective adaptation involving the repeated bout effect and the role of BCAA supplementation in this context. Thus, future studies should try chronic BCAA supplementation (and even other amino acids) against placebo with the same nitrogen load (isonitrogenous supplementation protocol) in order to evaluate the possible impact on muscle functionality and relate such effects with molecular pathways involved in muscle repair and regeneration.

## List of abbreviations

α- KIC: alpha-ketoisocaproate; ATP: adenosine triphosphate; BCAA: branched-chain amino acids; BCKDH: branched-chain alpha-keto dehydrogenase complex; CK: creatine kinase; DOMS: delayed onset muscle soreness; LDH: lactate dehydrogenase; MHC: myosin heavy chain; RE: resistance exercise.

## Competing interests

The authors declare that they have no competing interests.

## Authors' contributions

CRL participated in manuscript design, wrote the first draft of the manuscript. HN, NEZ, and DFSC participated in the interpretation and preparation of the manuscript. AHL Jr participated in manuscript design, interpretation and preparation of the manuscript. All authors read and approved the final manuscript.
